# FISH Going Meso-Scale: A Microscopic Search for Chromatin Domains

**DOI:** 10.3389/fcell.2021.753097

**Published:** 2021-11-03

**Authors:** Antonina Maslova, Alla Krasikova

**Affiliations:** Laboratory of Nuclear Structure and Dynamics, Cytology and Histology Department, Saint Petersburg State University, Saint Petersburg, Russia

**Keywords:** chromatin domains, chromatin imaging, fluorescence *in situ* hybridization (FISH), FISH probes, fluorescent microscopy, topologically associating domains, genome compartments, Oligopaints

## Abstract

The intimate relationships between genome structure and function direct efforts toward deciphering three-dimensional chromatin organization within the interphase nuclei at different genomic length scales. For decades, major insights into chromatin structure at the level of large-scale euchromatin and heterochromatin compartments, chromosome territories, and subchromosomal regions resulted from the evolution of light microscopy and fluorescence *in situ* hybridization. Studies of nanoscale nucleosomal chromatin organization benefited from a variety of electron microscopy techniques. Recent breakthroughs in the investigation of mesoscale chromatin structures have emerged from chromatin conformation capture methods (C-methods). Chromatin has been found to form hierarchical domains with high frequency of local interactions from loop domains to topologically associating domains and compartments. During the last decade, advances in super-resolution light microscopy made these levels of chromatin folding amenable for microscopic examination. Here we are reviewing recent developments in FISH-based approaches for detection, quantitative measurements, and validation of contact chromatin domains deduced from C-based data. We specifically focus on the design and application of Oligopaint probes, which marked the latest progress in the imaging of chromatin domains. Vivid examples of chromatin domain FISH-visualization by means of conventional, super-resolution light and electron microscopy in different model organisms are provided.

## Introduction: Overview of the Toolkits for Chromatin Domain Imaging

The term “chromatin” was coined by Walther Flemming at the end of the 19th century to designate structures stained by aniline dyes and confined within the cell nucleus (Paweletz, 2001). Together with significant improvements in sample preparation, detailed observations of chromatin behavior during cell division in different organisms and tissues made by Flemming, Walter Sutton, Karl Rabl, Theodor Bovery, and many other famous cytologists were possible through using light microscopes with state-of-the-art lenses, corrected for spherical and chromatic aberrations (Coleman, 1965). In the chromatin research timeline, the development of new microscopes and microscopy techniques together with chemical, biochemical, and later molecular biology methods for chromatin “contrasting” or “labeling” marked important milestones and defined the research trends for decades. The bulk of the methods, making chromatin details visible via a microscope, could be roughly classified into two categories: those that reveal protein components of the chromatin (mainly histones) and those that focus on DNA (reviewed in Lakadamyali and Cosma, 2015; Shao et al., 2017; Xu and Liu, 2019). Our current view on chromatin organization in the nucleus is drawn by implements from both protein and DNA detection. In this review, we will mainly concentrate on the evolution of DNA targeting techniques and tools, which fueled the recent success in imaging of subchromosomal chromatin domains at genomic length scales from several kilobases (kb) to several megabases (Mb). This most elusive “mesoscale” level of higher-order chromatin organization, mainly dissected by chromosome conformation capture technologies, is becoming open for microscopic examination.

At the beginning of the journey toward understanding the chromatin structure, conventional light microscopy was the only way to directly observe chromatin and chromosomes both in the nucleus and in spread (Cremer and Cremer, 2006). Interphase chromatin had been more readily detected when stained with common histological dyes and by Feulgen reaction, but finer details, other than the most intensely stained regions of chromosomes, remained indiscernible. Intensively stained heterochromatin was persistent and visible throughout the cell cycle in contrast to euchromatin, which decondensed in the interphase (Heitz, 1929). Large masses of heterochromatin were generally observed at the nuclear periphery, near the nucleolus and in chromocenters (Barr and Bertram, 1949; Hsu et al., 1971). Despite limited instruments, early works provided the first evidence for large-scale chromatin structures and their non-random distribution within the nucleus (Comings, 1968). Later, fluorescent DNA dyes came into use as a straightforward and simple way to stain the chromatin and to unravel cell type-specific differences in its spatial arrangement (Latt, 1977; Ellison and Howard, 1981; Agard and Sedat, 1983; Solovei et al., 2009; Berchtold et al., 2011).

Chromatin ultrastructure at a nanometer scale has been intensively investigated by electron microscopy, which achieved a resolution three orders of magnitude higher than light microscopy (Woodcock and Horowitz, 1997; Daban, 2011). It was estimated that 10 nm chromatin fibers could account for only 6 fold linear DNA packaging, which forced the research toward deciphering other “levels” of higher-order chromatin compaction (Fussner et al., 2011). Transmission electron microscopy of thin sections of nuclei revealed chromatin filaments of larger size (from 30 to 130 nm) populating the nuclear volume and dense heterochromatic areas near the nuclear envelope and nucleolus (Belmont et al., 1989; Kuznetsova and Sheval, 2016). When interpreting chromatin structures, it should be borne in mind that chromatin compaction is highly sensitive to surrounding conditions (Albiez et al., 2006; Maeshima et al., 2019). For this reason, chromatin images taken by transmission electron microscopy are frequently criticized for possible artifacts caused by harsh sample preparation, including dehydration, contrasting with heavy metals, resin or plastic embedding and ultrathin sectioning (van Holde and Zlatanova, 1995; Mielańczyk et al., 2015). Efforts toward the preservation of chromatin ultrastructure and its nuclear environment stimulated the development of cryo-electron microscopy (Dubochet et al., 1988), serial microtome block-face scanning electron microscopy (Rouquette et al., 2009), focused ion beam milling combined with scanning electron microscopy (Hoang et al., 2017) and other techniques. Novel method of chromatin contrasting for electron microscopy, called ChromEM, can be effectively coupled with electron microscopy tomography (ChromEMT; Ou et al., 2017), transmission electron microscopy (ChromTEM) (Li et al., 2021) and scanning electron microscopy (ChromSTEM) (Huang et al., 2020; Li et al., 2021). ChromEMT demonstrated that in interphase nuclei and mitotic chromosomes chromatin is packed into disordered 5–24 nm granular chain with highly variable folding parameters and packing density.

Absence of underlying genetic sequence information remains a main obstacle for detailed investigation of the mesoscale chromatin domains, identified by electron microscopy. Indeed, when examining the ultrastructural image of chromatin, specific chromosomal regions are unidentifiable (Woodcock and Ghosh, 2010). Nowadays, to discern the ultrastructural organization of a certain genomic region, electron microscopy is combined with the identification of specific genomic sequences.

Fluorescent microscopes and the first prototypes of confocal laser scanning microscope came into emergence in the first half of the 20th century (Renz, 2013). However, their expansion and wide implication in chromatin studies started in 70th due to the appearance of a critical method, overcoming “sequence specificity” problem. Mary-Lou Pardue and Joseph Gall showed that DNA probes could effectively hybridize with complementary target DNA sequences in cytological preparations (Pardue and Gall, 1969). This technology, named nucleic acids *in situ* hybridization (ISH; John et al., 1969), transformed into fluorescence *in situ* hybridization (FISH), when hapten (or dye)-modified nucleotides and fluorescent streptavidin or antibody detection had been widely applied instead of radioisotope labeled probes (Langer-Safer et al., 1982; Manuelidis et al., 1982; Pinkel et al., 1986; Wiegant et al., 1991). Evolution in probe design, fluorochrome diversity, and versatility of labeling protocols made ISH compatible with practically any microscopy technique including transmitted light microscopy, fluorescent, laser-scanning confocal and super-resolution light microscopy, as well as electron and correlation microscopy (Hutchison et al., 1982; Manuelidis, 1984; Rouquette et al., 2010; Weiland et al., 2011; Markaki et al., 2012; Jahn et al., 2016). Synergy of FISH and microscopy allowed to investigate individual gene loci and higher order genome organization relative to different nuclear compartments (Marshall, 2002), revived and expanded the theory of chromosome territories (Lichter et al., 1988; Cremer and Cremer, 2010), moved forward concepts of the dynamic nature of spatial genome organization and its close interdependence to genome functional state (Chubb and Bickmore, 2003; Kosak and Groudine, 2004; Misteli, 2005; Pueschel et al., 2016).

While FISH reveals DNA component of chromatin in fixed cells, immunofluorescent staining generally aims to detect chromatin proteins. Labeling of specific histone modifications, chromatin-associated proteins and components of chromatin-remodeling complexes allowed localizing structural and functional chromatin domains. Given the complexity of genome regulatory pathways, the development of FISH and immunofluorescent staining is directed to detect and visualize multiple targets in one experiment including the combination of FISH and immunofluorescent staining on the same preparation and automation of basic experimental procedures (Lin et al., 2015; Shachar et al., 2015; Huber et al., 2018). Before addressing FISH tools for mesoscale chromatin domain imaging, we will briefly focus on the delineation of these genome compartments by chromatin conformation capture.

## Capturing an Image of Chromatin Domains

The emergence of the chromosome conformation capture (3C) technique in the first decade of the 21st century on the basis of nuclear ligation assay (Cullen et al., 1993) and the subsequent expansion of 3C technology had a great impact on the field of 3D genomics (Dekker et al., 2013; Denker and de Laat, 2016). 3C-derivate methods (Hi-C, 5C, ChIA-PET, Micro-C, etc.) allowed to dissect three-dimensional genome organization with resolution and throughput, unattainable by other approaches based on imaging (Fraser et al., 2015b; Goel and Hansen, 2020). Moreover, they facilitated the understanding of the functional significance of identified spatial genome folding due to the alignment of Hi-C data with other genome-wide landscapes (Sati and Cavalli, 2017). However, during the last decade, improving sequencing depth and “high throughput” power of C-based methods and the concurrent development of bioinformatics tools for the analysis of complex and big data have led to some ambiguities in the interpretation of results and terminological confusion (Marti-Renom et al., 2018; Ing-Simmons and Vaquerizas, 2019; Pal et al., 2019). The key initial steps of C-experiments – crosslinking and proximity-based ligation – were also pointed out as potential sources of biases and limitations (Gavrilov et al., 2015; Kempfer and Pombo, 2020). While recent updates in both data analysis and experimental procedures challenge some of the biases (Brant et al., 2016; Belaghzal et al., 2017, 2021), the ability of C-methods to capture multimodal interactions of genomic loci, differentiate stable from short-lived interactions and examine cell-to-cell contact variability is still limiting (McCord et al., 2020).

**FIGURE 1:**
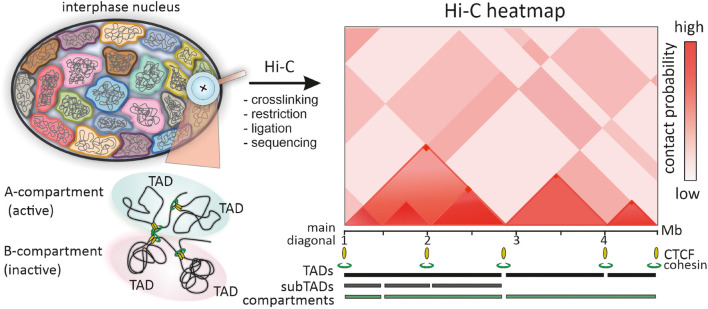
Chromatin domains identified by Hi-C. In Hi-C procedure chromatin of interphase chromosomes is cross-linked and restricted, closely positioned genomic loci are ligated and sequenced by high-throughput sequencing. The frequency of pairwise contacts of genomic loci is represented in the squire matrix or color-coded genomic interaction Hi-C heatmap, which is symmetric along the main diagonal (one halve is shown for simplicity). The A/B compartment segregation is reflected in checkboard-like pattern of the Hi-C heatmap. TADs are identified along the main diagonal of the heatmap as mega- to sub-megabase scale triangle domains with enriched interactions of genomic loci within a domain. Nested subTADs shown within the first TAD are the main features of mammalian Hi-C heatmaps. Corner-dots represent loops between TAD borders, mediated by CTCF and cohesin.

Color-coded heatmaps of pairwise interactions of genomic loci, a common form of data presentation in high-throughput C-methods, provide information on intra- and interchromosomal interactions at different genomic length scales from dozens of megabases down to ∼200 bp (Ing-Simmons and Vaquerizas, 2019; Mota-Gómez and Lupiáñez, 2019; Figure 1). Resolution of these maps is important for the interpretation of Hi-C data. For example, early low-resolution Hi-C maps of the human genome demonstrated chromatin segregation into multi-megabase-sized compartments – active (A) and inactive (B) (Lieberman-Aiden et al., 2009; Figure 1). Higher resolution genomic heatmaps combined with epigenetic and transcriptomic data allowed to characterize finer subcompartments – two for A type (A1 and A2) and four for B type (B1, B2, B3 and B4) (Rao et al., 2014).

Another distinctive feature of Hi-C heatmaps in many species are local contact domains, known as topologically associating domains (TADs; Dekker and Heard, 2015). Since the first description of TADs in 2012, numerous facets of TAD structure, mechanisms of formation, dynamics during the cell cycle and ontogenesis, functional implications in gene regulation and genome folding have been deeply studied (Dixon et al., 2016; Szabo et al., 2019; Beagan and Phillips-Cremins, 2020). Paradoxically, the more data on TADs are accumulated, the more difficult it is to give a unified definition of TAD (de Wit, 2020). In the initial heatmaps of mammalian and *Drosophila* genomes, TADs were defined as diagonal domains of variable size (∼ 60 kb for *Drosophila* and ∼880 kb for mammals), where genomic loci belonging to the same TAD show higher interaction frequency than loci assigned to neighboring TADs (Dixon et al., 2012; Hou et al., 2012; Nora et al., 2012; Figure 1). Thus, the borders of TADs restrict genomic interactions between domains. However, even from these early maps it was clear that TADs comprise smaller substructures (subTADs), and the observed high-frequency contacts arise via chromatin looping (Figure 1). As with compartments, the development of Hi-C technology toward higher heatmap resolution enabled to detect finer details including discrete loops, insulated neighborhoods, enhancer-promoter contacts, etc. (Dowen et al., 2014; Rao et al., 2014; Krietenstein et al., 2020). TADs frequently appeared as assemblies of a number of nested domains and included into larger super-structures called TAD-cliques and meta-TADs (Fraser et al., 2015a; An et al., 2019; Collas et al., 2019). This hierarchy was also revealed by many computational domain-calling tools, but with variable correspondence in the identified domain borders (Weinreb and Raphael, 2016; Forcato et al., 2017; Zufferey et al., 2018). The matter of TAD detection is further complicated by the fact that some TADs, subTADs and loop domains arise via cohesin-mediated loops between convergent CTCF sites, manifested by off-diagonal “corner dots,” while the other TADs and loop domains do not (Dixon et al., 2016; Beagan and Phillips-Cremins, 2020; de Wit, 2020; Figure 1). Unified classification of TADs and self-interacting domains could simplify comparison of data from multiple studies and orthogonal approaches, including microscopy.

While the functional significance of TADs in gene regulation has convincing experimental evidence, the physical nature and chromatin structural counterparts of TADs remain enigmatic (Szabo et al., 2018). Some data indicated that TADs could scarcely represent stable units of chromatin organization and appear in population-average Hi-C heatmaps as a mere statistical manifestations of different permitted chromatin conformations regulated by architectural proteins at the level of individual cells (Giorgetti et al., 2014; Dixon et al., 2016). It was clear that these issues could not be resolved by C-method alone and needed aid from microscopy-based methods. The demand of visualization of small neighboring genomic regions has stimulated the development of chromatin imaging by FISH approaches with broad involvement of confocal and super-resolution optical microscopy (Boettiger and Murphy, 2020; Cardozo Gizzi et al., 2020).

## Hi-C – Fish Paradox

From the very beginning of TAD studies, microscopy and FISH-based approaches were used as complementary methods to verify the patterns and “structures” seen in Hi-C heatmaps (Nora et al., 2012; Sexton et al., 2012; Rao et al., 2014). Intuitively, two genomic loci that exhibited higher contact frequency when analyzed by C-based approaches should be found closer in a nuclear space as determined by distance measurement. The inverse correlation between loci contact frequency and 3D-distance was indeed observed in a number of studies (Nora et al., 2012; Giorgetti et al., 2014). However, few loci escaped this correlation, showing longer distances at high-interaction frequency sites (Williamson et al., 2014). Somewhat contradictory results from FISH-based and Hi-C methods provoked discussion in the field on how the data from these two orthogonal approaches could be cross-validated and reconciled (Dekker, 2016; Giorgetti and Heard, 2016; Fudenberg and Imakaev, 2017). As was noted, “Hi-C – FISH paradox” emerges from the intrinsic variability in physical proximity between two linearly distant genomic loci in single cells, so that their direct contact is a relatively rare event, still registered as a significant interaction by Hi-C in a million cell population (Finn et al., 2019; Shi and Thirumalai, 2019).

From this point of view, high-throughput power of Hi-C turns into a “drawback” as this approach is not able to predict chromatin folding and interactions in a particular cell nucleus. To overcome this limitation, significant efforts have been made in the development of single cell Hi-C and complementary techniques (Ulianov et al., 2017). What appeared from single cell Hi-C experiments is that individual pairwise interactions from different cells were highly variable. At the same time, cumulative heatmaps obtained from dozens or hundreds of analyzed cells generally recapitulated the patterns of conventional Hi-C maps (Nagano et al., 2013; Ramani et al., 2017; Stevens et al., 2017). Additionally, the “Hi-C – FISH paradox” could be solved by high-throughput FISH-imaging of multiple genomic loci in hundreds of cells, followed by averaging of pairwise distances between loci and generation of distance proximity matrices, which could be directly compared to Hi-C contact heatmaps (Boettiger and Murphy, 2020; Hu and Wang, 2021). Moreover, C-methods generally capture very close genomic contacts within nuclear space, which is defined by the paraformaldehyde crosslinking radius (presumably 10–100 nm). Thus, to obtain a better correlation between FISH and Hi-C data one could look in a range of distances that are clearly beyond the diffraction limits of the conventional optical microscopy. FISH data obtained by super-resolution microscopy and specific probes demonstrate very close concordance with C-data (Szabo et al., 2020).

## Fish as a an Efficient Approach for Chromatin Domain Visualization

The efficiency of FISH, as a direct metlhod of DNA-target visualization in fixed cells and tissues, relies on the targeting capacity of the probe and fluorescent dyes used for direct probe labeling or indirect probe detection (Beatty et al., 2002). FISH probes could detect targets from individual genes (few kb) to whole chromosomes and genomes. However, due to the high linear DNA packaging ratio in the interphase nuclei (1:300–1:3000), most individual genes are under the resolution limits of conventional fluorescent microscopy (∼200–250 nm) (Lawrence et al., 1990). This means that morphological details and distance measurements below this range are difficult and inconsistent, as any object would appear as a blurred point due to the diffraction of light. Visualization of genomic regions smaller than ∼10 kb is particularly demanding and usually requires extensive probe design or signal amplification (Schriml et al., 1999; Rogan et al., 2001; Yamada et al., 2011; Beliveau et al., 2015; Ni et al., 2017).

Another resolution-related and genome compaction issue is a genomic distance between simultaneously visualized genomic regions. Directly neighboring small genomic regions detected by FISH probes coupled to different dyes would apparently appear as one spot of co-localized signals. Depending on the epigenetic status of the visualized region (extended euchromatic or compact heterochromatic) 10–50 kb of linear genomic distance is required to discriminate regions as individual signals within the interphase nucleus to allow accurate 3D-distance measurements (Yokota et al., 1997). Thus, the rationale behind probe design is a key to comparative studies between Hi-C and FISH and to the visualization of structures seen in Hi-C maps. In further sections, we are reviewing the most common probes used for verification of C-based data and recent innovations in probe design.

While, theoretically, many probes could be used for simultaneous hybridization, due to the limited number of detection fluorochromes and the architecture of imaging systems, most often 1–3 genomic targets are visualized at one round of hybridization during FISH experiment. This major limitation of FISH is partly solved by applying combinatorial fluorochrome schemes for probe detection resulting in mixed colors or repeated sequential hybridization of samples with probes of interest (Ried et al., 1992; Hu and Wang, 2021). The current progress of FISH throughput in both the number of simultaneously detected probes and the number of cells analyzed in one experiment is tightly linked to the development of automated microfluidic systems (Huber et al., 2018). Apparently, probe and microscopy choice depends on the purposes of a particular experiment, the resolution and throughput that should be obtained (Gelali et al., 2018).

**FIGURE 2:**
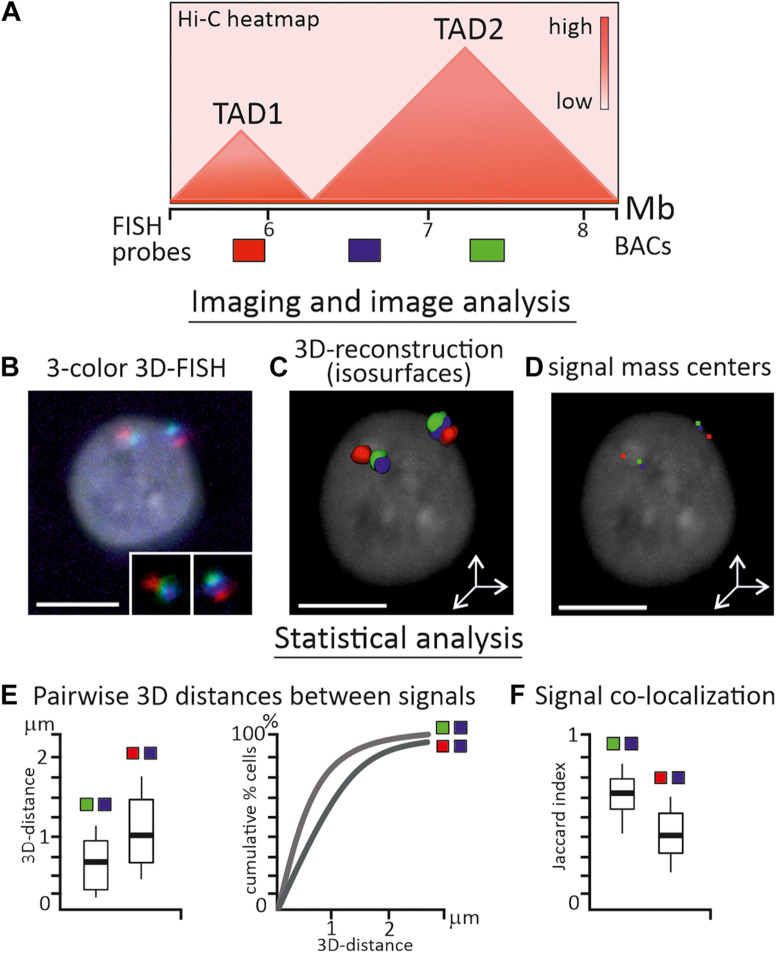
Analysis of TAD properties by FISH-visualization of genomic regions. **(A)** Schematic heatmap of two neighboring TADs in chicken genome and relative positions of three BAC-based FISH probes, which are separated by ∼500 kb from each other. **(B)** Confocal microscopy image (maximum projection) of chicken interphase nucleus after 3-color 3D-FISH with BAC-based FISH probes, FISH signals from two homologous chromosomes are enlarged. **(C)** 3D-surface reconstruction of FISH-signals using Imaris (Oxford Instruments) software. **(D)** Locations of signal mass centers (red, green and blue pixels) are calculated from the reconstruction. **(E)** Main statistics is inferred from pairwise 3D-distance measurements between mass centers of imaged regions in a cell sample and could be presented as boxplots or cumulative graphs. **(F)** Signal co-localization statistics is estimated by Pearson correlation coefficient (not shown) or Jaccard index of signal overlap, depicted as boxplot. Probes from the same TAD (green-blue pair) usually exhibit shorter 3D-distances and higher overlapping, than probes from neighboring TADs (red-blue pair). Nucleus is counterstained with DAPI. Scale bars, 5 μm.

For verification of medium to low resolution Hi-C-derived data within and between megabase-sized contact domains, the resolution of conventional laser-scanning confocal microscopy is sufficient (Figures 2A,B). However, when assessing chromatin conformation within TADs, subTADs, and particular loop domains, one should consider methods of super-resolution fluorescent (SRM) or electron microscopy. In the last decade, stochastic optical reconstruction microscopy (STORM), photoactivated localization microscopy (PALM), structured illumination microscopy (SIM), focused ion beam scanning electron microscopy (FIB-SEM) were applied intensively in the exploration of chromatin domains within individual cells (Lakadamyali and Cosma, 2015; Birk, 2019; Szydlowski et al., 2019; Shim, 2021; Xie and Liu, 2021). Highlights and practical guidance for the application of these microscopy tools is beyond the scope of the present review and can be found elsewhere (Lambert and Waters, 2017; Schermelleh et al., 2019).

A microscopic image is the main source of information for imaging-based methods; therefore the requirements for image acquisition, equipment settings and image analysis pipelines should be particularly strict (Ronneberger et al., 2008). To obtain reliable data from fine-scale image analysis, possible distortions in the image and systematic errors should be carefully considered, eliminated or correctly adjusted (Ronneberger et al., 2008). The frequency of pairwise genomic loci interactions from C-data could, with some reservations discussed above, be correlated with the distances between the pair of loci from image-based data (Figures 2B–D). As such, the main statistics during image analysis were inferred from direct 2D or 3D-distance measurements between loci in a number of individual cells (Finn et al., 2017; Figure 2E). Independently of the form of the observed signal, i.e., dot-like or more extended irregular-shaped objects, usually program-assisted segmentation of thresholded signals is applied followed by calculation of centroid coordinates – a proxy of locus nuclear spatial position (Szabo et al., 2021; Figures 2C,D). Co-localization analysis between signals is also frequently performed and in some cases is more informative than distance distribution analysis, especially when FISH-targeted regions are genomically close or consecutive (Giorgetti and Heard, 2016; Figure 2F). As two-color and multicolor images may suffer from chromatic aberrations of optical systems, the chromatic shift between channels should be estimated and corrected in distance measurements (Kozubek and Matula, 2000). Custom-made scripts, plugins in free image software, and commercial packages are effectively implemented for image adjustment, object segmentation, and analysis. In experimental pipelines where multiple loci are imaged in hundreds of cells, image acquisition, error correction and measurements are fully automated (Su et al., 2020). Wide spectrum of other characteristics could be estimated during statistical analysis of FISH-images, including signal density, volume and 3D-shape, gyration radius of a signal, scaling exponent of power law dependence of genomic to physical distance over an extended genomic region or a chromosome, clustering of loci and proximity to nuclear landmarks, etc. (Boettiger et al., 2016; Wang et al., 2016; Liu et al., 2020; Szabo et al., 2021). The inclusion of microscopy-derived parameters together with C-method-derived parameters in data-driven polymer models of chromatin were shown to enhance the modeling results (Abbas et al., 2019).

## Fish-Probes for Chromatin Domain Visualization

### Clone-Based Probes

**FIGURE 3:**
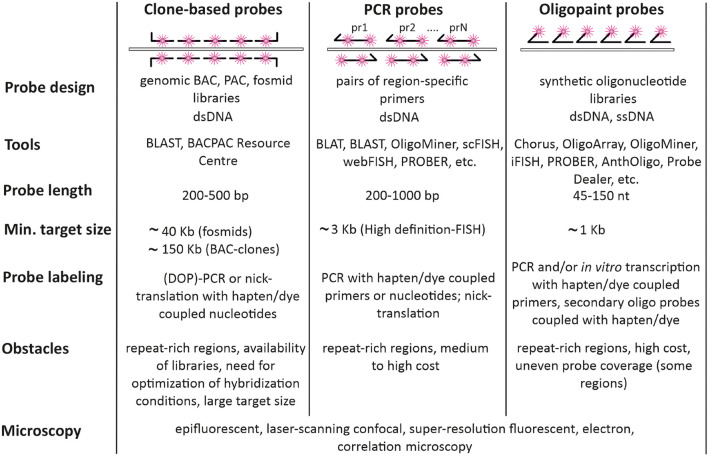
Types of probes used for FISH-visualization of Hi-C chromatin domains. Clone-based probes, PCR probes, and Oligopaint probes are compared in terms of design, labeling, and complications.

Probes, based on cloned sequences (PACs, BACs, fosmids, etc.) are the most common FISH probes and have been intensively used in interphase cytogenetics and genome architecture studies from the 1980th (Landegent et al., 1987; Figure 3, left column). BAC-clones contain inserts of genomic DNA of a particular species in a range of 50–300 kb, large enough for reliable visualization with fluorescent microscopy. Labeled probes are generated from BAC DNA via enzymatic incorporation of modified nucleotides (conjugated with either hapten or fluorochrome) during nick translation, (DOP)-PCR or whole-genome amplification, including rolling-circle amplification (Bayani and Squire, 2004; Sharma and Meister, 2020). The enzymatic labeling produces shorter probes with a high labeling density of 20–40 dye/hapten-modified nucleotides per kb (Yu et al., 1994). Both the large size of the target and the high density of labels produce strong FISH signals needed for precise quantitative measurements Figures 2B–D. However, the quality of the clone-based probes greatly relies on the performance and activity of the enzymes used in the enzymatic labeling as different polymerases incorporate modified nucleotides with variable efficiency (Tasara et al., 2003; Anderson et al., 2005). Likewise, the density of probe labeling depends on the structure of the nucleotide-fluorochrome complex and fluctuates significantly, when different fluorochromes are utilized for the same enzymatic reactions (Zhu et al., 1994; Giller et al., 2003). This limitation is partially solved by applying two-step enzymatic probe labeling, when at the first step amynoallyl-modified nucleotides (5-(3-aminoallyl)-2′-deoxyuridine 5′-triphosphate) are efficiently incorporated into the probe DNA by polymerases and at the second step amino-reactive dyes (for example N-hydroxysuccinimide esters of fluorochromes) are used for binding to amino-groups (Cox and Singer, 2004; Bolland et al., 2013). Alternatively, enzyme-free nucleic acid labeling kits with platinum dye complexes are commercially available.

Being originated from relatively large genomic fragments, clone-based probes may exhibit moderate specificity due to the presence of repetitive sequences (Sealey et al., 1985). When this is the case, the overall specificity of the probes could be increased by adding preannealing of the labeled probe with competitor DNA (Cot-1 DNA) – the fast-renaturing repetitive DNA fraction of the same species, which is needed to suppress repetitive sequences within a probe (Lichter et al., 1988). Unique sequences of clone-based probes could be also enriched by Cot-1 and duplex-specific nuclease-assisted removal of repetitive sequences before enzymatic labeling (Swennenhuis et al., 2012). Considering above-mentioned issues, the protocols of labeling and FISH with particular clone-based probes may require significant efforts toward optimization. While BAC and PAC libraries with large genome coverage are available for many species (see, for example),^1^ for some they are sparse or absent. Moreover, in poorly assembled genomes BAC-contigs could be placed incorrectly leading to the need for additional verification of chromosomal position for any particular BAC-clone. In summary, clone-based probes remain the probes of choice for imaging of relatively large genomic regions by both conventional microscopy and super-resolution microscopy due to the probe robustness, relatively low cost, versatile labeling and detection protocols.

### PCR-Derived Probes

When the sequence of the DNA region to be visualized by FISH is known, FISH probes could be produced directly from genomic DNA via PCR with specific primers (Figure 3, middle column). Amplified products could be labeled by PCR. In this case, hapten, fluorochrome, or amine-modified nucleotide is added to the reaction. PCR amplicons could be also labeled by adding modified nucleotides with terminal deoxynucleotidyl transferase or by nick translation. PCR-generated probes are widely applied for repeated genomic targets (centromere and telomere repeats, ribosomal genes, etc.). However, PCR-generated probes for single-copy genomic targets of several kb in size may require enhanced detection protocols involving signal amplification (Bayani and Squire, 2004). One of the strategies is tyramide signal amplification, which allows identification of targets less than 1 kb (Schriml et al., 1999). This procedure has certain limitations when several targets are visualized simultaneously. Moreover, non-linear and hardly controlled signal amplification for multiple targets may compromise resolution and quantitative methods like co-localization analysis. PCR-generated probes, which are more than 1 kb in size, may require size optimization to improve penetration into the cell, which is achieved by probe digestion with restriction enzymes. Another straightforward strategy for increasing the visibility of single-copy genomic targets is to cover the whole region by smaller probes, produced by PCR with multiple pairs of primers. However, careful bioinformatic analysis of the region and primer selection should be done to exclude amplification of interspersed repeats. A number of tools for picking primers for PCR labeling have been suggested, including PROBER (Navin et al., 2006), webFISH (Nedbal et al., 2012), and scFISH (Rogan et al., 2001; Figure 3). For human and mouse genomes, there is a database of specific primers covering the whole genome but omitting repeated sequences. By using these primers, it is possible to produce FISH-probes from 100 to 200 bp amplicons with a density of 80 amplicons per 100 kb (Bienko et al., 2013; Gelali et al., 2018). This high-definition FISH (HD-FISH) allows detecting 3 kb targets without signal amplification (Bienko et al., 2013).

### Oligonucleotide Probes and Oligopaints

Oligonucleotides arrived on the FISH scene with the development of automated oligonucleotide synthesis. However, their use was limited to identifying repetitive sequences, spanning large genomic regions and usually found within the centromere, telomere, and nucleolus organizer regions of chromosomes (Matera and Ward, 1992). Presently, oligonucleotide probes are the probes of choice to detect genomic regions down to several kb, which are clearly smaller than the typical inserts in BAC clones (Figure 2, right column). Moreover, this type of probe performs equally well when used to visualize large targets from extended gene loci to whole chromosomes (Boyle et al., 2011; Jiang, 2019).

**FIGURE 4:**
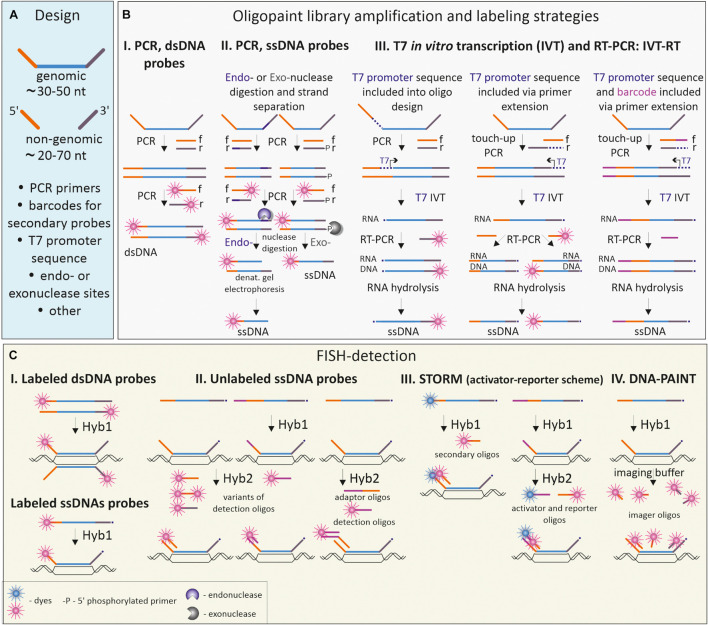
Oligopaint probe design, labeling, and detection schemes. **(A)** Basic design of an oligo from the Oligopaint library. Each oligo comprises a DNA sequence complementary to the genomic DNA of the species of interest and non-genomic 5′- and 3′-flanking regions (frequently referred to as the mainstreet and backstreet correspondingly), needed for amplification, barcoding and detection of Oligopaints. **(B)** Common strategies for labeling and modification of Oligopaint libraries. **(B.I)** Double-stranded Oligopaint probes (dsDNA, labeled or unlabeled) are obtained by PCR with forward (f) and reverse (r) primers. **(B.II)** Low-to-medium quantities of single-stranded probes are generated by introducing a recognition site for an endonuclease in one of the non-genomic flanking regions and subsequent PCR. Endonuclease makes a nick in one strand of the amplified dsDNA; the desired DNA strand is isolated and purified by denaturation gel electrophoresis. Alternatively, PCR with 5′-phosphate labeled primers (-P) could be used to introduce sites for Lambda exonuclease, which digests the 5′-phosphate labeled strand. **(B.III)** High quantities of single-stranded Oligopaint probes are generated by sequential PCR, *in vitro* transcription and reverse transcription (IVT-RT). T7 promoter sequences could be included either in the Oligopaint design within the mainstreet region downstream to the primer sequence or added via “touch-up PCR” with one of the primers bearing T7 promoter sequence. Barcodes with different functionalities (for example, sequences for secondary detection oligos) could be also added to the mainstreet or backstreet via PCR with barcoded primers. **(C)** FISH-targeting and detection of double-stranded (dsDNA) and single-stranded (ssDNA) Oligopaint libraries using one-round probe hybridization **(C.I)** or two-round hybridization with detection or adaptor/detection oligos **(C.II)**. Detection schemes could be adapted to implement fluorescent super-resolution microscopy such as STORM (C.III) or PAINT (C.IV).

During the last decade, the development of cost-effective techniques for massive parallel oligo synthesis and accumulation of genome sequencing data have boosted the application of oligonucleotide-based FISH-probes and culminated in the development of Oligopaint technique (Beliveau et al., 2012). Oligopaints, pools of tens of thousands of oligonucleotides, have high specificity, controlled complexity and enable a versatile design matching various detection schemes and microscopy applications (Beliveau et al., 2017). The basic design of the Oligopaint includes a region complementary to the genome target tagged with non-genomic sequences at the 3′ and 5′ ends (Figure 4A). At a minimum, these non-genomic tags contain sequences for primers, required to amplify (and sometimes simultaneously label) the entire Oligopaint library, since after synthesis the concentration of any certain oligonucleotide in the pool is usually low (femtomoles) (Murgha et al., 2014). Oligopaint 3′ and 5′ tag regions could be extended to comprise several primers allowing amplification of a certain oligo sublibrary, which may be useful and cost-effective when differential labeling of several smaller regions within a larger one is needed. Moreover, the non-genomic tags could be extended with additional sequences, depending on the labeling and detection schemes applied in a certain experiment (Figure 4A). Several strategies were introduced for the amplification, labeling, and modification of Oligopaint probes depending on the size of the visualized region, nature of the visualized target (DNA, RNA, or both) and species (Figure 4B; Beliveau et al., 2012; Chen et al., 2015; Wang et al., 2016; Fields et al., 2019).

The most straightforward method to obtain double-stranded Oligopaint probes from the synthesized library is to amplify the whole library with labeled primers, complementary to tag regions of Oligopaints (Beliveau et al., 2012; Figure 4B.I). This approach proved to be productive for the generation of whole chromosomal paints or probes to large chromosomal regions (Bi et al., 2020). Amplified double-stranded Oligopaint probes could be transformed into single-stranded form via introducing the nuclease digestion site into non-genomic tag for subsequent nicking, followed by denaturation and gel purification of the desired strand (Figure 4B.II; Beliveau et al., 2012, 2015; Murgha et al., 2014). However, when large amounts of single-stranded labeled probe are needed, a more convenient procedure has been introduced (Murgha et al., 2014; Chen et al., 2015). In this case, the whole library of Oligopaints is first amplified, transcribed *in vitro*, and then reverse-transcribed with specific labeled primers (see IVT-RT on Figure 4B.III). To direct enzymatic reactions, the sequences of the PCR-primers and T7 promoter for RNA polymerase should be included into the non-genomic tags of the Oligopaints either during *in silico* design or via touch-up PCR to the already amplified oligo libraries (Figure 4B.III). The IVT-RT method and its modifications have become widely applied to amplify and label Oligopaint libraries in different species from plants to human (Boettiger et al., 2016; Gelali et al., 2019; Jiang, 2019).

The recognized advantage of Oligopaint-based probes is the flexibility of design in terms of detection issues (Figure 4C). Fluorochrome or hapten-labeled primer(s), complementary to one or both tag-regions, are used during PCR amplification or *in vitro* transcription to obtain labeled oligonucleotides and visualize the target in one round of hybridization (Figure 4C.I). Another scheme relies on using 2-step hybridization: the first one with an unlabeled Oligopaint probe and the second one with dye-coupled detection oligo which is complementary to the tag sequences of Oligopaint (Beliveau et al., 2015; Gelali et al., 2018; Fields et al., 2019; Figure 4C.II). The second scheme is more versatile as it allows changing the color for visualization of the same probe by changing the label of the detection oligo, without the need of relabeling the entire library. It is widely applied when multiple locus-specific Oligopaint probes are visualized in the same cell (Wang et al., 2016; Cardozo Gizzi et al., 2019; Fields et al., 2019). Complex detection schemes with activator and photoswitchable reporter dyes should be taken into consideration during design of the Oligopaint libraries for high-resolution imaging of small-scale genomic targets by single-molecule localization microscopy (SMLM) methods, like STORM (Figure 4C.III) or DNA point accumulation for imaging of nanoscale topography (DNA-PAINT; Figure 4C.IV; Boettiger et al., 2016; Beliveau et al., 2017; Bintu et al., 2018; Nir et al., 2018).

Construction of Oligopaint FISH probes for a certain genomic region starts with the generation of a database of oligonucleotides, tiling the primary sequence, and subsequent optimization of the oligonucleotide pool to ensure the needed specificity and complexity of the probes (Beliveau et al., 2012). There lay few pitfalls hindering the application of Oligopaints. Regions with high repeat content, erroneously or poorly assembled, could hardly be unambiguously covered by oligos and are excluded. Moreover, the application of Oligopaints is confined to popular model organisms with well-assembled genomes. Another difficulty concerns an extensive bioinformatic expertise to decide on the essential parameters needed to filter the initial oligonucleotide pool, even though multiple tools for designing custom arrays of oligonucleotides for genomic regions have been suggested, including OligoArray (Rouillard et al., 2003), PROBER (Navin et al., 2006), Chorus (Han et al., 2015), OligoMiner (Beliveau et al., 2018), iFISH (Gelali et al., 2019), AnthOligo (Jayaraman et al., 2020), ProbeDealer (Hu et al., 2020). The array-synthesized Oligopaint probes are expensive, compared to BAC- or PCR-based probes for the same-sized genomic regions; however, the cost of probes per hybridization could be comparable if high-throughput FISH is performed (see for discussion Beliveau et al., 2012; Boettiger and Murphy, 2020). Among many other parameters used for optimization of the Oligopaint library, the density of oligos per kb should be thoroughly streamlined. It not only influences the size of the Oligopaint library and therefore its cost, but also specifies the reliability of target detection during FISH. As a general principle – the smaller the target region to be visualized, the denser oligo coverage of the region is required to obtain a robust FISH-signal. Practically, the highest density of 15–20 oligos/kb is needed to detect regions from several kb to several dozen kb (Beliveau et al., 2012; Gelali et al., 2019), 10–15 oligos/kb sufficiently detect regions from one to several Mb, while only 0.1–5 oligos/kb are shown to be enough for dozen Mb-sized regions or whole chromosomal paints (Han et al., 2015; Rosin et al., 2018; Jiang, 2019; Bi et al., 2020).

In summary, FISH probes, based on pools of *in silico* designed synthetic Oligopaint libraries progressively displace clone-based probes in experimental designs where high-throughput single cell visualization of multiple and small genomic regions with maximal resolution is required (Figure 2). FISH with Oligopaint probes is an extremely rapidly evolving field in terms of Oligopaint design for multiplying the number of simultaneously targeted regions, protocol adjustments for detection of RNA and proteins, or both. For these reasons, Oligopaints are widely applied to assess the organization of TADs, A/B compartments and other chromatin domains in different model organisms (Boettiger and Murphy, 2020; Hu and Wang, 2021). Moreover, this type of FISH-probe is used to address questions of chromatin fiber organization within highly compacted metaphase chromosomes (Kubalová et al., 2021). In further sections, we briefly review major insights from FISH-imaging, which together with 3C-based methods aided pieces to the puzzle of spatial organization of chromatin domains in human, mouse, *Drosophila* and other model genomes.

## Fish-Visualization of Chromatin Domains in Model Organisms

### Visualization of Chromatin Domains in Mammals

**FIGURE 5:**
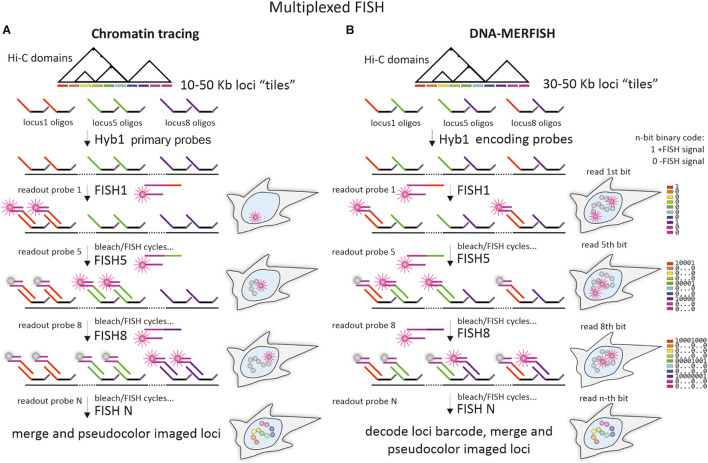
Multiplexed imaging of chromatin domains by chromatin tracing and DNA-multiplexed error-robust FISH. **(A)** The pipeline of chromatin tracing techniques with one-color/one locus in a round (shown for simplicity) and “adaptor oligo/detection oligo” detection scheme. A whole library of Oligopaint probes (primary library), targeting one or several chromatin domains, is divided into multiple sublibraries of oligos with unique mainstreet regions. After initial hybridization of primary probes, individual loci with locus-specific mainstreets are detected during sequential rounds of hybridization with locus-specific readout probes. Design of the adaptor oligo, containing regions, complementary to locus-specific mainstreets and to detection oligos, implements the detection scheme, where a universal dye-labeled detection oligo could be used for sequential imaging of all individual loci. Alternatively, mainstreet-specific dye-labeled oligos could be used (not shown on a scheme). To ensure detection of a particular locus at a time, readout probes are photobleached after imaging or washed out during the subsequent FISH-detection round by using additional small oligonucleotides for strand-displacement reactions (not shown on the scheme). **(B)** The pipeline of DNA-multiplexed error-robust FISH (DNA-MERFISH). A whole library of Oligopaint probes (encoding probes), targeting chromatin domains is divided into multiple sublibraries of oligos, bearing locus-specific “barcodes,” encoded in the unique combination of mainstreet and/or backstreet regions (for simplicity shown by different colors of mainstreet regions only). After initial hybridization of the encoding probe, the individual units (bits) of the locus-specific barcodes are detected during sequential rounds of hybridization with “bit”-specific readout probes (one-color detection with reading one bit at a time is shown) until all bits are detected. To ensure a detection of a particular “bit” at a time, dyes are removed or photobleached after imaging. Imaging of all loci in chromatin tracing and decoding of all locus-specific barcodes allow to identify the spatial coordinates of individual loci and to reconstruct chromatin “folding” within the domain. Generally, due to the combinatorial format of individual locus identification, fewer rounds of readout hybridization are required for imaging of the same number of loci in DNA-MERFISH, compared to chromatin tracing.

In the first papers, conceptualizing A/B compartments and TADs, FISH was used to visualize regions belonging to contact domains in mouse and human cell lines (Lieberman-Aiden et al., 2009; Dixon et al., 2012; Nora et al., 2012). Since then FISH-based visualization has become a “gold standard” not only to verify C-based data but to analyze the spatial architecture of particular chromatin domains (Giorgetti and Heard, 2016; Bintu et al., 2018). FISH signal evaluation gives an opportunity to test various domain properties (for instance, self-confinement, border-insulation, or large-scale association) within the nuclear context in a single cell. 3D distances between linearly equidistant genomic targets are shorter when measured in the same TAD than between neighboring TADs in numerous regions analyzed so far (Nora et al., 2012). Moreover, BAC-probes covering the whole TAD or several sequential TADs, such as those in the mouse *HoxD* gene cluster, were often discerned as separate globular domains, still variable in shape (Fabre et al., 2015). For *HoxD* gene cluster, it was also shown that the overall morphology of either the extended or more compacted *HoxD* regions does not necessarily correlate with the transcriptional state of the locus (Fabre et al., 2015). FISH-targeting of genes and regulatory sequences belonging to TAD within a α*-globin* locus in mouse embryonic stem cells (mESCs) and differentiating erythroblasts showed that distinct domain shapes and specific *cis*-contacts are established before transcriptional activation (Brown et al., 2018). Systematic studies assessing 3D-distances between BAC-probes to the same or different TADs across many chromosomal regions clearly demonstrated the local variability of chromatin folding at the level of TADs in individual cells (Finn et al., 2019). As it was shown for TADs, regions belonging to the same compartment tended to be closer in a nuclear space (Lieberman-Aiden et al., 2009). However, while these studies illuminated the spatial organization of particular genomic loci, they still gave little idea on the cytological equivalents of various contact domains. Recently, many questions regarding the presence of TADs, their physical parameters, spatial organization, and segregation of A/B compartments *in cis* and *in trans* as well as their relation to other nuclear domains, were addressed directly due to development of novel chromatin imaging technologies enabling tracing of the chromatin paths within the nucleus (Hu and Wang, 2021; Figure 5).

Chromatin tracing combines Oligopaint probes, multiplexed FISH-imaging of dozens or even hundreds of small genomic loci within a contiguous chromosome segment, and image analysis tools to visualize the chromatin paths from the subTAD to chromosomal level in individual cells (Figure 5A). Chromatin tracing also allows measuring pairwise spatial distances between multiple imaged loci and constructing heatmap matrices (both for a single cell and averaged between thousands of cells) similar to contact frequency maps in Hi-C or 5C. These heatmaps reflect the mean distances between the imaged loci or the frequency of their proximity (Wang et al., 2016; Bintu et al., 2018; Nir et al., 2018; Su et al., 2020). Interestingly, on such distance- and proximity maps, generated for several chromosomes (20, 21, 22, X) in IMR90 human lung fibroblasts, there were distinct compartments that highly correlated with A and B compartments on Hi-C maps (Wang et al., 2016; Su et al., 2020). While the relative spatial distribution of loci belonging to different compartments varied between cells, the tendency for segregation of loci into A and B higher order domains were clearly observed. Similarly, in GM23248 human skin fibroblasts, super-resolution microscopy demonstrated that segments of the same type of compartments within the ∼8 Mb region on chromosome 19 clustered together and consisted of more distinct chromatin bundles (Nir et al., 2018).

Recent coupling of the chromatin tracing technology with RNA-multiplexed error-robust FISH (RNA-MERFISH) and immunofluorescence staining made it possible to simultaneously visualize chromosomal loci, RNA and nuclear domains (for example, nucleoli and lamina) in mouse embryonic liver cells (Liu et al., 2020) and human IMR90 cells (Su et al., 2020). This opened up wide opportunities to relate transcription and chromatin compartmentalization the individual genomic regions. As it was shown for mouse chromosome 19, its distance-derived compartment profile differed among certain liver cell types. Significant rise in the expression of genes harbored in particular TADs was associated with an increase in A-to-B compartment ratio of the locus; however, the increase in A-to-B ratio itself was not mandatory for changes in gene expression (Liu et al., 2020). In human cells, A-to-B ratio was higher around transcribed genes, nascent transcripts of which were visualized along chromosome 21 together with their chromatin walk (Su et al., 2020). Genome-wide examination of 50 kb loci and transcripts of their genes also showed that genes experiencing high transcriptional activation resided in A compartment (Su et al., 2020). In this latter state of the art study, introducing DNA-MERFISH method (Figure 5B), concurrent imaging and analysis of more than a thousand loci from all human chromosomes demonstrated extensive *trans* interactions of the loci. More specifically, interchromosomal and long-range intrachromosomal contacts occurred preferentially between genomic loci belonging to A-compartment, while short-range intrachromosomal contacts (in a scale below 70 Mb) occurred preferentially between genomic loci belonging to B-compartments. Multiple loci imaging also confirmed earlier observations on enrichment of genomic loci belonging to B-compartments near the nuclear lamina and nucleolus, and enrichment of genomic loci belonging to A-compartment in proximity to nuclear speckles (Chen et al., 2018; Quinodoz et al., 2018).

Taking into account the almost decade-long extremely intense investigation of genome architecture by C-methods, possibly the most relevant question to ask is whether TADs could be captured by microscopy as chromatin domains somehow insulated from neighboring chromatin? Several recent studies involving chromatin tracing of 5–50 kb regions and both super-resolution and diffraction-limited fluorescent microscopy succeeded in quantitative imaging of chromatin conformation at the level of TADs and other contact domains (Bintu et al., 2018; Nir et al., 2018; Liu et al., 2020; Su et al., 2020; Beckwith et al., 2021). TAD-like chromatin domains of variable size, compactness, and degree of segregation were indeed observed in single cells (Bintu et al., 2018; Su et al., 2020). By analogy with population-averaged TADs, spatial distances between the foci within microscopically identified single-cell chromatin domains were shorter than between foci of neighboring domains. However, the borders separating one single-cell domain from the others were not permanent and their positions fluctuated from cell to cell (Bintu et al., 2018; Su et al., 2020). Surprisingly, these single-cell chromatin domains were insensitive to cohesin removal (Bintu et al., 2018). Another study used DNA stains, SIM, electron, and correlation microscopy to visualize the chromatin network and utilized denaturation-free RASER (resolution after single-strand exonuclease resection)-FISH to map the positions of several TADs against chromatin substructures (Miron et al., 2020). As appeared, TADs could fall into chains of delineated chromatin nanodomains of 200–300 nm in size, which were also resistant to cohesin ablation (Miron et al., 2020).

Highly similar results were obtained in an independent study that addressed the internal organization of TADs and utilized Oligopaint-based super-resolution imaging of individual TADs in mESCs (Szabo et al., 2020). The authors suggested that chromatin nanodomains are true physical subunits of TADs, the formation of which is largely stochastic (i.e., variable number of nanodomains per TAD in single cells). Indeed, the histone deacetylase inhibitor trichostatin A disrupted chromatin nanodomains, changing their volume and number (Szabo et al., 2020). While the size range and other properties of single-cell chromatin domains revealed by chromatin tracing and chromatin nanodomains observed by microscopy generally overlaps, how the two types of domains relate to each other remains to be elucidated.

Nevertheless, chromatin domain profiles, essentially corresponding to TAD profiles in Hi-C maps, emerged after averaging spatial distance (or proximity frequency) matrices between hundreds of cells (Bintu et al., 2018; Su et al., 2020). Furthermore, borders of the “averaged” single cell chromatin domains were marked by CTCF and cohesin binding sites and were sensitive to cohesin depletion. These experiments clearly showed that in individual cells chromatin folding at the level of TADs was highly variable, but certain regions exhibited higher probability of domain border formation. Some evidence indicates that the relationship between TADs and compartments in mammalian genomes is more complex than simply hierarchical and that the formation of TADs and compartments are guided by different mechanisms (Mirny et al., 2019). In line with this, it was shown that apart from single-cell chromatin domains of “pure” A- or B compartment type, a significant number of single-cell domains comprised different proportions of both types (Su et al., 2020). How compartmentalization communicates with loop extrusion-assisted TAD formation remains an expanding field of research.

Heterogeneity of chromatin-folding structures within distinct loop domains has also been demonstrated in human cell line by ISH combined with serial block-face scanning electron microscopy (3D-EMISH) (Trzaskoma et al., 2020), FISH with interferometric PALM (iPALM; Jufen Zhu et al., 2019) and loop tracing with DNA-PAINT in non-denaturing conditions (Beckwith et al., 2021). In the 3D-EMISH study, BAC-probes covering the 1.7-Mb region on chromosome 7 in lymphoblastoid GM12878 cells were detected with 1.4-nm-thick streptavidin-conjugated fluoronanogold, followed by analysis of density center distribution in reconstructed ultrastructural serial images of targeted chromatin region. While BAC-probes used in this study could not discriminate between three distinct CTCF-bordered loop domains, identified by ChIA-PET (Chromatin Interaction Analysis by Paired-End Tag sequencing) within this region, 3D-EMISH generally captured from one to four microscopically identified domains, which had highly variable structure and volume (Trzaskoma et al., 2020). Similarly, imaging-based models of a single 13 kb loop and its 10 kb flanking regions in T-cell receptor alpha locus, probed by FISH and iPALM, showed multiple loop conformations in single cells. Still, the pairwise distances for most of the conformations reproduced inverse correlation between frequency of interactions and distance when compared with Hi-C and ChIA-PET data (Jufen Zhu et al., 2019).

A recent study, combining non-denaturing RASER-FISH, DNA-PAINT and chromatin tracing at single-loop scale (kb to Mb region near the *Myc* locus), confirmed some conclusions from a loop extrusion model of TAD formation (Beckwith et al., 2021). Specifically, while the folding of chromatin fibers is intrinsically random and variable from cell to cell, structural elements in CTCF-bound sites interfere with chromatin fiber random coiling and organize chromatin into loops and self-interacting domains, seen in cell population averages.

From impressive studies of chromatin domains by direct imaging it can be suggested that when the scope is shifted from population-based observations to single cells, stable patterns appear blurred and chromatin contacts – variable. While being spotted as compact globular chromatin domains, mammalian TADs and the loops that build them are something other than deterministically persistent structures with stable borders. Structural variability of chromatin organization could be linked to the variability of functional outcome and adds another level to genome regulation.

### Visualization of Chromatin Domains in *Drosophila*

**FIGURE 6:**
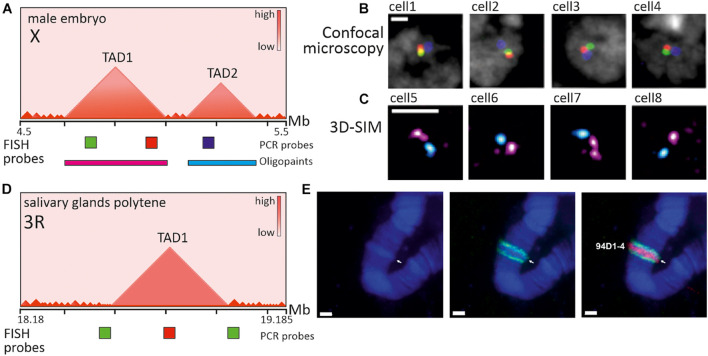
Imaging of TADs in Drosophila interphase cells and polytene chromosomes. **(A)** Schematic heatmap of two large consecutive TADs from X chromosome in male embryos and relative positions of PCR-derived and Oligopaint FISH probes. **(B)** Confocal microscopy images (z-slices) of several nuclei after 3-color FISH with genomically equidistant probes (red, green, blue), used for inter-probe 3D distance measurement (not shown) and verification of contacts within and between TADs (adapted with permission from Figure 3B in Szabo et al., 2018). In most cells probes from one TAD are closer in a nuclear space, than probes from neighboring TADs. **(C)** 3D-SIM images (maximum projections) of several nuclei after 2-color FISH with Oligopaint probes (magenta and cyan), targeting TAD1 and TAD2 correspondingly (adapted with permission from Figure 3F in Szabo et al., 2018). In most nuclei two large TADs appear as distinct globular nanocompartments. **(D)** Schematic heatmap of a region with one large TAD from chromosome 3R in salivary gland cells of wandering third instar larvae and relative positions of PCR-derived FISH probes. **(E)** Images of polytene chromosome 3R (fragment) after 2-color FISH with probes covering TAD center (red) and TAD borders (green) (adapted with permission from Figure 4C in Eagen et al., 2015). Probe to TAD is observed within a band, indicated by arrow and identifier, while probes to border regions are in the flanking interbands. Nuclei and chromosomes are counterstained with DAPI (B, gray; E-blue). Scale bars: **(B,C)** –1 μm, **(E)** –2 μm.

Being for more than a century a model organism in genetic laboratories, the fruit fly *Drosophila* has the most thoroughly characterized genome among invertebrates (Tweedie et al., 2009; Jennings, 2011). *Drosophila* genome, examined by C-methods in different cell types, possesses both TADs and compartments (Hou et al., 2012; Sexton et al., 2012; Rowley et al., 2017). This fact led to the conclusion that these levels of chromatin folding could be common for Metazoa. However, in some respect, *Drosophila* TADs are clearly distinct from mammalian ones. Notably, domain borders and contacts within TADs in *Drosophila* generally do not rely on looping interactions promoted by CTCF and cohesin (Ulianov et al., 2016; Matthews and White, 2019). In *Drosophila*, TAD formation is rather driven by transcription (Hou et al., 2012; Rowley et al., 2017), histone modifications (Sexton et al., 2012; Ulianov et al., 2016; El-Sharnouby et al., 2017), insulator elements and bound proteins (Ramírez et al., 2018; Wang et al., 2018; Bag et al., 2021). Along the *Drosophila* genome, large repressed TADs generally alternate with active regions (also known as boundary regions, inter-TADs or active TADs), occupied by smaller contact domains (subTADs, mini-domains) (Rowley et al., 2017; Ramírez et al., 2018; Wang et al., 2018; Figure 6A). A collective term “compartment domains” was coined for all identified *Drosophila* contact domains due to the correlation of TAD profile with epigenetic profiles and clear segregation of contact domains into two types, corresponding to either A or B compartments (Rowley et al., 2017).

Given the small size of both *Drosophila* genome and TADs/compartment domains themselves, efficient visualization of chromatin domains in the interphase nucleus has been achieved by super-resolution microscopy. In correspondence with the emerging picture of epigenetically specified compartment domains, microscopic observations identified repressed TADs as distinct nanometer-sized domains and active regions as extended chains of dotted subdomains (Cattoni et al., 2017; Szabo et al., 2018; Figures 6A–C). According to the spatial parameters of chromatin folding deduced from imaging, repressed TADs demonstrate a higher degree of chromatin compaction (Boettiger et al., 2016; Szabo et al., 2018). Interestingly, knockdown of several PcG-proteins, associated with repressed H3K27Me3 epigenetic domains in *Drosophila*, led to partial disassembly of repressed domains to more open conformations, indicating the role of PcG-proteins in domain maintenance (Boettiger et al., 2016).

Two cognate approaches to chromatin tracing combined with RNA detection (Hi-M and ORCA) allowed to visualize several adjacent TADs in *Drosophila* embryos (Cardozo Gizzi et al., 2019; Mateo et al., 2019). Multiplexed sequential imaging approach (Hi-M) allowed tracing of 21 loci within the ∼350 kb region of two TADs, one of which contained genes essential for development and expressed during the early stages of zygotic genome activation. Averaged distance proximity maps constructed for this region highly correlated with Hi-C maps and followed the pattern of mitotic disappearance and reappearance of TADs during zygotic genome activation. Transcriptional activation of genes within microscopically identified TADs leads to perturbation of TAD structure (Cardozo Gizzi et al., 2019). In another study, optical reconstruction of chromatin architecture (ORCA) tiled 100–700 kb regions of the *bithorax* complex (*BX-C*) in several differentiating cell types of *Drosophila* embryos, discriminated by simultaneous mapping of 30 RNA species. This approach allowed to track cell type specific changes in the microscopically identified TAD patterns in *BX-C* locus and to correlate them with changes in epigenetic status accompanied by transcription activation. Upon sequential activation of *BX-C* genes, the inactive TAD substantially contracted and its boundary moved to the right, separating the still inactive H2K27Me3 and PcG-rich chromatin from the active regions. Importantly, smaller TADs appeared within the active region, bounding distinct genes and their regulatory regions. The borders of these TADs were independent of PcG activity, but contained CTCF and CP190, indicating that CTCF may also play a role as an insulator of contact interactions at least in some of the chromatin domains in *Drosophila* (Mateo et al., 2019).

Apparently, the most illustrative “cytological” interpretation of TADs as structural chromatin domains is the banded pattern of polytene chromosomes in *Drosophila*. Early light and electron microscopy observations of polytene chromosomes from salivary glands and other tissues demonstrated that dense (black and gray) bands and more diffuse interbands alternate along the length of these extended interphase chromosomes (Kolesnikova, 2018). Persistent morphology of polytene chromosomes gave birth to the idea that certain principles of interphase chromosome folding can be encoded in polytene “barcodes” (Vatolina et al., 2011). Indeed, Hi-C of polytene chromosomes disclosed TADs as conserved genome architectural features between polytene and conventional somatic cell nuclei (Eagen et al., 2015). Substantial overlapping of TADs with polytene bands and interTAD regions with interbands has been demonstrated (Eagen et al., 2015; Ulianov et al., 2016; Stadler et al., 2017). FISH probes to TAD borders and internal regions perfectly mapped to interbands and bands correspondingly (Eagen et al., 2015; Figures 6D,E). Thus, *Drosophila genome* is characterized by a large degree of correlation between TADs/compartment domains seen in population-averaged Hi-C maps and microscopically visualized chromatin nanodomains in individual cells, as well as by correlation between TADs/compartment domains and polytene bands. In line with this conclusions, drawn from imaging, single-cell Hi-C of *Drosophila* BG3 cell line showed that ∼40% of TADs are conserved between individual cell nuclei (Ulianov et al., 2021). In comparison, in mammals, single-cell TADs are highly variable and comprise multiple nanodomains. Whether this discrepancy in nanoscale chromatin folding between *Drosophila* and mammals could be attributed to the loop extrusion mechanism or the basic differences in genome size and distribution of regulatory elements and genes remains to be elucidated. In this respect, imaging of chromatin topology in various species with differing genome structures seems to be of great value.

### Visualization of Chromatin Domains in Other Model Organisms

In Hi-C heatmaps of other representatives of Vertebrata, including fish (*Danio rerio)* (Kaaij et al., 2018), amphibians (*Xenopus tropicalis)* (Niu et al., 2021) and birds (*Gallus gallus)* (Fishman et al., 2019), compartments, TADs and loops are readily discerned. Avian chromatin domains are of special interest in terms of FISH-visualization since certain cell types demonstrate dramatic changes in 3D-genome organization. Indeed, during chicken erythropoiesis, typical TADs disappear, while long-range interactions between distant genomic loci come into place (Fishman et al., 2019). Moreover, similar to polytene chromosomes found in *Drosophila* ovarian nurse cells, avian growing oocytes bear giant transcriptionally active lampbrush chromosomes with a distinct chromomere-loop structure (Gaginskaya et al., 2009). FISH-based approaches are now applied to establish a correspondence between meiotic lampbrush chromomeres and chromatin domains in the interphase nucleus (Krasikova et al., 2019; Zlotina et al., 2020).

TAD-like self-associating domains and/or compartments have been found in other widely studied model organisms from different taxa, including yeasts (*Saccharomyces cerevisiae, Schizosaccharomyces pombe*), worms (*Caenorhabditis elegans*), plants and even prokaryotes (Rowley and Corces, 2016; Dong et al., 2017). These TAD-like domains, being much alike in appearance (triangles along the diagonal in Hi-C maps), are highly variable in size, chromosomal distribution, and functional significance and may be shaped by diverse factors and mechanisms (Dekker and Heard, 2015; Rada-Iglesias et al., 2018; Dong et al., 2020). For example, in *C. elegans*, X chromosomes of XX hermaphrodite animals consist of self-interacting TAD-like domains. Boundaries of many of these self-interacting domains are occupied by the dosage compensation complex (DCC), a condensin placed on *rex* sites (Crane et al., 2015). FISH-probes flanking TAD boundaries confirmed the insulation property of *rex* sites, which was disrupted upon DCC loss (Crane et al., 2015).

Large-scale interphase organization of plant chromosomes, segregation of eu- and heterochromatic chromosomal regions and their positioning relative to nuclear landmarks, such as nucleolus or nuclear periphery, have been meticulously probed by FISH and microscopy in several model plants (Pecinka et al., 2004; reviewed in Schubert and Shaw, 2011). Plant chromosome territories in nuclei of different species and/or tissues could display a large variety of spatial conformations from Rabl configuration to plant-specific “Rosette” configuration (Rodriguez-Granados et al., 2016). FISH with BAC-clone derived probes showed that in *Arabidopsis* each chromosome territory forms compact heterochromatic chromocenter “core” surrounded by (sub)megabase-sized euchromatic loops (Fransz et al., 2002), which could be involved in long-range interactions (Schubert et al., 2014). Hi-C studies of *Arabidopsis*, rice (*Oryza sativa*), and maize (*Zea mays*) interphase chromatin, revealed an important role of repeat content in shaping local chromatin domains (Dong et al., 2020). In contrast to plants with large genomes, like rice, that exhibit TAD-like domains (Ouyang et al., 2020), *Arabidopsis* features few domain-like structures. At the same time, rice and *Arabidopsis* are characterized by additional functional long-range interacting domains that may be plant-specific – inactive heterochromatic islands (IHIs) or KNOT engaged Elements (KEEs). FISH with probes to IHIs confirmed that KNOT is formed by IHIs that could belong to different chromosomes (Feng et al., 2014; Grob et al., 2014). Oligopaints are intensively used in comparative plant cytogenetics for the development of chromosome-specific painting probes and loci-specific probe sets (Jiang, 2019), and therefore may serve as a promising tool for deciphering fine-scale spatial chromatin architecture in plant nuclei. Given the diversity and peculiar chromatin domain structures in plant species, the application of novel methods of chromatin imaging for plant genomes will be of high priority (Dumur et al., 2019).

## Conclusion and Perspectives

We are currently witnessing large leaps forward in microscopy tools and FISH-based techniques, which attained genomic and spatial resolution unimaginable just several years ago. As a reminiscence of evolution in C-methods, the demand of ever-growing genomic resolution and higher throughput stimulate the development of chromatin imaging toward multiplexing the number of visualized genomic loci together with other nuclear landmarks, increase in the number of cells analyzed, streamlining and unifying of protocol and analysis pipelines. Apart from further expansion of multiplexed sequential FISH (Xiao et al., 2020; Takei et al., 2021), recent coupling of Oligopaint probes for targeting genomic loci and fluorescent *in situ* sequencing (OligoFISSEQ) demonstrated the potential for imaging more targets in fewer rounds of sequencing and with higher resolution, than chromatin tracing and DNA-MERFISH (Nguyen et al., 2020). The gap between imaging-based and sequencing-based methods of spatial genome probing is progressively shrinking. Combination of chromatin imaging by immunofluorescent staining or fluorescent protein tags and Hi-C on the same single cell offers hope for direct juxtaposition of data, obtained on the genome in exactly the same conformation (Lando et al., 2018). However, the convergence of multiplexed high-resolution FISH and Hi-C in one experiment faces some difficulties. Heat denaturation, fixation, and permeabilization may disturb fine-scale chromatin structures (below 1 Mb) (Solovei et al., 2002; Markaki et al., 2012). Large efforts have been taken to develop probes for hybridization in more physiological and non-denaturing conditions, which maximally preserve the structure of small-scale chromatin domains (Hausmann et al., 2003; Schmitt et al., 2010). Among promising strategies are Cas9-mediated FISH (CASFISH; Deng et al., 2015), RNA-guided endonuclease *in situ* labeling (RGEN-ISL; Ishii et al., 2019) and RASER-FISH (Brown et al., 2018).

FISH, as Hi-C, is typically performed on fixed cells and tissues and captures only snapshots of the chromatin in action. Nevertheless, even in fixed cells, the observed heterogeneity of chromatin topologies at genomic length scales from tens kb to several Mb could mirror the constrained dynamics and plasticity of chromatin fibers (Hansen et al., 2018). Imaging of chromatin in living cells by expression of fluorescent protein-tagged histones revealed high dynamics and variability (in terms of size and shape) of multiple nucleosomal assemblages – chromatin “blobs,” speculatively corresponding to self-interacting domains on Hi-C maps (Wachsmuth et al., 2016; Nozaki et al., 2017).

Tracing of individual chromatin contact domains *in vivo* would allow more precise analysis of chromatin fiber behavior, domain persistence time, stochastic and specific interactions, and other parameters, which are crucial for understanding the principles behind chromatin domain formation. Attaining this goal is tightly linked to the development of probes for *in vivo* labeling of genomic loci (both artificially inserted and endogenous), generally based on the operator-repressor methods (LacO/LacI, TetO/TetR), ANCHOR/ParB system, transcription activator-like effectors (TALEs) or clustered regularly interspaced short palindromic repeats (CRISPR)/nuclease-deactivated CRISPR-associated protein 9 (dCas9) technology (reviewed by Eykelenboom and Tanaka, 2020). CRISPR/dCas9 technology utilizes single guide RNAs (sgRNAs) to target complementary DNA locus and recruit dCas9 fused with fluorescent protein toward the DNA/RNA duplex (Chen et al., 2013). CRISPR/dCas9-based technology evolves exceedingly fast in terms of adapted labeling strategies to enhance signal-to-noise ratio and single locus visibility within cell nucleus (Wu et al., 2019). For instance, multicolor and high-resolution live cell tracking of loci and monitoring of inter-loci distances was achieved using either three dCas9 with different sgRNA binding specificity (Ma et al., 2015) or by engineering sgRNA to harbor RNA aptamers, recognized by cognate binding proteins (Clow et al., 2020).

Rapid advances in chromatin imaging and the spreading of these “hi-end” techniques within the scientific community hold promise for decoding the mesoscale spatial and temporal organization of the genome and its multifaceted regulatory capacity in the near future.

## Author Contributions

AM wrote the original draft, prepared the figures, and edited the manuscript. AK suggested concept of the article, critically revised the manuscript, and the figures. Both authors approved the final version of the manuscript.

## Conflict of Interest

The authors declare that the research was conducted in the absence of any commercial or financial relationships that could be construed as a potential conflict of interest.

## Publisher’s Note

All claims expressed in this article are solely those of the authors and do not necessarily represent those of their affiliated organizations, or those of the publisher, the editors and the reviewers. Any product that may be evaluated in this article, or claim that may be made by its manufacturer, is not guaranteed or endorsed by the publisher.

## Footnotes

1
https://bacpacresources.org/

## Abbreviations

(DOP)-PCR: degenerate oligonucleotide-primed polymerase chain reaction
(F)ISH: fluorescence *in situ* hybridization
(i)PALM: (interferometric) photoactivated localization microscopy
3C: chromosome conformation capture
3D-EMISH: three-dimensional serial block-face scanning electron microscopy combined with *in situ* hybridization
5C: chromosome conformation capture carbon copy
BAC: bacterial artificial chromosome
ChIA-PET: chromatin interaction analysis by paired end tag sequencing
CRISPR/dCas9: clustered regularly interspaced short palindromic repeats/nuclease-deactivated CRISPR-associated protein 9
CTCF: CCCTC-binding factor
DNA-PAINT: DNA point accumulation for imaging of nanoscale topography
Hi-C: high-throughput chromosome conformation capture
Hi-M: high-throughput multiplexed sequential imaging
IHIs: inactive heterochromatic islands
IVT-RT: *in vitro* transcription and reverse transcription
MERFISH: multiplexed error-robust FISH
mESCs: mouse embryonic stem cells
Micro-C: micrococcal nuclease Hi-C
PAC: phage P1-derived artificial chromosomes
PcG: polycomb group
RASER-FISH: (resolution after single-strand exonuclease resection)-FISH
SIM: structured illumination microscopy
SRM: super-resolution microscopy
STORM: stochastic optical reconstruction microscopy
TAD: topologically associating domain.
